# An infant with asymptomatic hepatic granuloma probably caused by bacillus Calmette-Guérin (BCG) vaccination found incidentally at autopsy: a case report

**DOI:** 10.1186/1757-1626-1-337

**Published:** 2008-11-20

**Authors:** Yutaka Tajima, Rie Takagi, Tamiko Nakajima, Yoshihiko Kominato

**Affiliations:** 1 Legal Medicine and Molecular Genetics, Postgraduate School of Medicine, Gunma University, Maebashi, 371-8511, Japan

## Abstract

**Introduction:**

Bacillus Calmette-Guérin (BCG) is an attenuated strain of *Mycobacterium bovis*. Usually, systemic complications due to BCG vaccination are quite rare. However, since BCG is a live vaccine, there is still a possibility that it may cause an infection.

**Case presentation:**

Hepatic granuloma was found incidentally in an asymptomatic 5-month-old infant who was found dead in his bed. The probable cause of death was asphyxia due to milk aspiration into the lungs. The granuloma was composed of epithelioid histiocytes with frequent multinucleated Langhans-type giant cells and a small number of lymphocytes.

**Conclusion:**

The cause of the asymptomatic granuloma was not identified, but was considered likely due to BCG vaccination.

## Introduction

Bacillus Calmette-Guérin (BCG) is an attenuated strain of *Mycobacterium bovis *(*e.g.*, Tokyo172 strain), which is still widely used as a vaccine (immunogen) against *M. tuberculosis *infections and is a potent immunomodulator. Usually, BCG vaccination causes only localized and weak skin irritation around the inoculation spots, and systemic complications are quite rare. However, since BCG is a live vaccine, there is still a possibility that it may cause an infection. Dissemination is a rare but well-known complication of BCG vaccination. It occurs principally in immunodeficient patients, and skin, bone, and visceral organs are sometimes affected. Less frequently, disseminated BCG infection has also been described in apparently normal (immunocompetent) patients [1,2]. Most previously reported cases have been in patients with bladder cancer who have undergone immunotherapy with BCG (intravesical installation) [3-8].

Here we report an infant in whom an asymptomatic hepatic granuloma was found incidentally at autopsy.

## Case presentation

A 5-month-old male infant (height 68 cm, weight 5.8 kg) was found dead in his bed. Further information concerning this event was not available. He had been born at full term of pregnancy (38 weeks) with a birth weight of 2.6 kg. His growth and development had been normal, and his parents were not aware of any physical abnormality. His body temperature and appetite had been normal until his death, and there were no relevant signs or symptoms. The infant had died after normal breastfeeding and being left to sleep. When his mother checked how he was, she found him dead in a prone posture on the bed. The bedclothes were soiled by a white and soft coagulated material, suggesting that the infant had vomited milk. He had been vaccinated with BCG 2 months before death. His family members all gave a normal tuberculin reaction, and there was no evidence that any of them suffered from active tuberculosis.

## Pathological findings

A complete autopsy was performed 26 h after the infant's death. Besides routine hematoxylin and eosin (H&E)-stained sections, additional sections were used for acid-fast bacilli staining and DNA extraction [9]. Polymerase chain reaction (PCR) for mycobacterium detection (Amplicor; Roche Diagnostics; Basel, Switzerland) was performed in accordance with the manufacturer's technical insert (lower detection limit = ~5 DNA copies/sample) [10].

Macroscopic examination revealed almost no visible abnormalities in any of the organs. Localized, small areas of weak skin irritation with scabs were around the BCG inoculation spots on the left arm.

**Figure 1:**
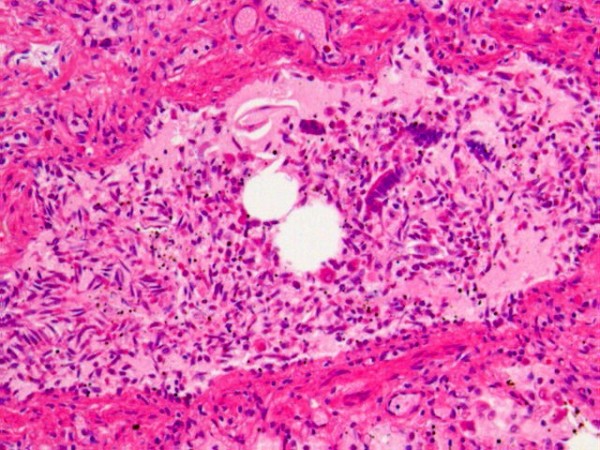
**Pathological findings in the lungs**. A number of bronchioli and alveolar ducts were obstructed with uneven eosinophilic material containing oral epithelium, alveolar macrophages, and massive bronchial epithelia (H&E, × 100).

Microscopic examination (Fig. 1) revealed that a number of bronchioli and alveolar ducts were obstructed with uneven eosinophilic material containing oral epithelium with bacterial growth, bile components, alveolar macrophages, and massive bronchial epithelia (including basal cells). As far as could be observed, most of the bronchial epithelium had peeled off with slight bleeding in the submucosal layers.

**Figure 2:**
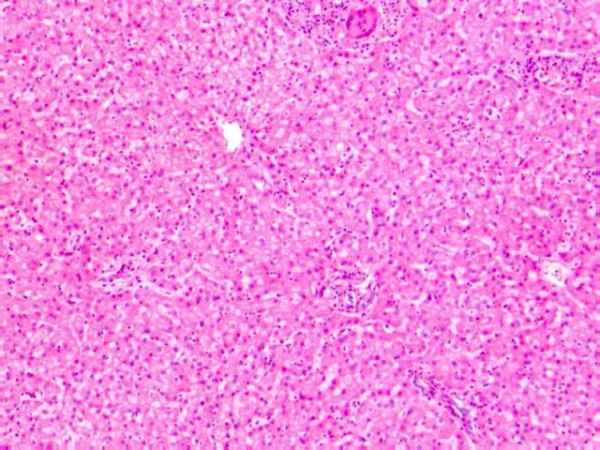
**Pathological findings in the liver**. Granulomas were found with no additional pathological changes (H&E, × 50).

**Figure 3:**
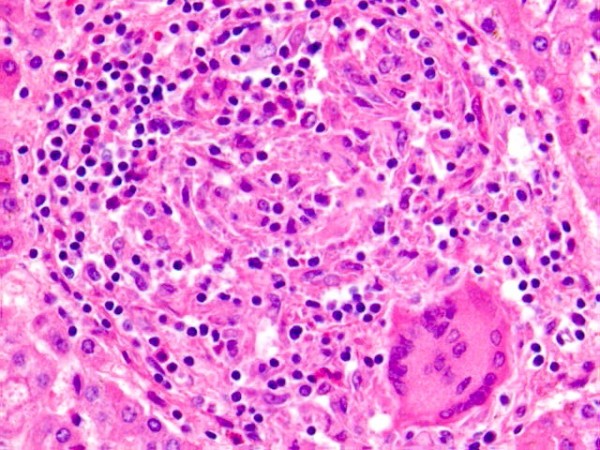
**Pathological appearance of the liver granulomas**. The granulomas were composed of epithelioid histiocytes with frequent multinucleated Langhans-type giant cells and a small number of lymphocytes. All granulomas were round in shape, clearly delimited, and well organized with no caseous necrosis (H&E, × 200).

Interestingly, liver tissue sections (Fig. 2) contained a number of microscopic granulomas (~10 per section), although no similar lesions were found in other organs, and otherwise the liver revealed no additional pathological changes. The granulomas found in the liver (Fig. 3) were composed of epithelioid histiocytes with frequent multinucleated Langhans-type giant cells and a small number of lymphocytes. All the granulomas were round in shape, clearly delimited, and well organized. No evidence of necrosis was found at the center of each granuloma, and an extensive inspection showed that none of the granulomas contained acid-fast bacilli or fungi. Sensitive PCR analyses performed on DNA extracts from paraffin sections were all negative for mycobacterium. In addition, we checked several similar cases submitted for autopsy (*i.e.*, infant sudden death after BCG vaccination), but no such granulomatous lesions were found.

## Discussion

Microscopic observation revealed that most of the epithelium in the respiratory tract had peeled off with submucosal bleeding. Usually, such findings are observed in patients who have received artificial ventilarory support, but this infant had not been resuscitated. Considering that milk aspiration (stomach contents) had probably occurred in the lungs, one possible interpretation of this finding is that the infant had exhibited strong labored breathing (or coughing) for a while to expel the aspirated liquid. Therefore, we considered that the cause of death had probably been asphyxia rather than sudden infant death syndrome (SIDS).

The presence of epithelioid cell granuloma in the liver usually indicates a diagnosis of disseminated BCG infection (miliary BCG) or classical miliary tuberculosis. The latter was ruled out, however, because of the absence of tuberculous lesions in other organs (*e.g.*, lungs, mediastinal lymph nodes, spleen, and meninges). In addition, the absence of acid-fast bacilli in histological sections associated with the negative mycobacterial PCR result indicated that this was not active infection due to mycobacterium. The possibility of sarcoidosis is rather remote in such a young infant [11], and there was no evidence suggesting this disease in the lungs. The absence of any additional pathological changes in the liver ruled out neonatal (giant cell-type) hepatitis and biliary tract diseases. Therefore, we considered that the hepatic granuloma had been induced by BCG vaccination.

Vaccination with BCG is obligatory for newborns in Japan, the first dose usually being given at 3~6 month of age. Although many reliable studies have demonstrated its beneficial effects and safety [12], BCG vaccination is not always without complications (Table 1) [13]. For example, a recent report has estimated the prevalence rate of symptomatic disseminated BCG to be below one per 10^6 ^vaccinations [14].

**Table 1:** Known complications of BCG vaccination

Local
Skin ulceration
Regional lymphadenitis (known as BCGitis)

Systemic
Immunocompetent (probably)
Disseminated skin eruption
Miliary granuloma (mainly lungs and liver)
Osteomyelitis
Immunocompromised
Severe systemic dissemination (with poorly formed granulomas and numerous bacilli)

As is the case with most infectious diseases, the host immunity seems to play an important role in the development of such complications. Disseminated infection is known to be one of the most serious complications of BCG vaccination, and occurs principally in immunocompromised hosts [1,15-17]. In such cases, a number of inflammatory lesions (*e.g.*, epithelioid cell granulomas) develop systemically (mainly in the lungs and liver). These granulomas are characterized by extensive tissue necrosis, poor structural formation, and extensive collections of histiocytes and epithelioid cells with abundant acid-fast bacilli, and only a few giant cells. The outcome is usually fatal, and autopsy reveals many visible lesions in almost every organ [1].

In Japan, there have been only two fatalities attributed to BCG vaccination, and both of the affected individuals were found to have immunodeficiency. The BCG strain used in Japan (Tokyo172 strain) is known to be highly safe for immunocompetent hosts. In healthy animals, BCG inoculation sometimes (frequently at a high dose vaccination dose) induces the formation of epithelioid cell granulomas (especially in the liver and spleen) [18], but most of them are only transient: *i.e.*, they will usually resolve without anti-mycobacterial chemotherapy (manufacturer's personal communication).

In immunocompetent humans, most BCG complications (if present) are local and/or regional (Table 1). However, disseminated BCG infection does occur rarely in apparently immunocompetent infants and children [19-24]. These patients show a well-developed granulomatous response with few (or no) bacilli inside the lesions, and their clinical outcomes are generally good. Our experience in the present case was rather similar, and therefore we considered that the hepatic granuloma had been due to transient and subtle BCG infection. Historically, Gormsen has also reported granulomas in forensic autopsy cases (*i.e.*, patients who had died due to unexpected causes such as trauma) [25]. According to his report, definite evidence of granuloma was not found in any organ later than 40 months after vaccination. In 13 of the 20 cases studied from 6 weeks to 40 months after BCG vaccination, granulomas were demonstrated in the liver (n = 10), lung (n = 6), peribronchial lymph nodes (n = 2), spleen (n = 2), and kidney (n = 2), but acid-fast bacilli were detected only in the regional lymph nodes near the vaccination site (not in other organs). In 11 cases the number of granulomas was small, whereas they were numerous in the other two which were low-weight infants of less than 1 year of age [25].

It has been demonstrated that BCG can be highly pathogenic to specific individuals for unknown reasons [9,20]. Since clinically symptomatic BCG dissemination has recently been interpreted as always resulting from an underlying immunodisorder (although this seemed to be clear in only 50% of the cases reviewed [2]), miliary BCG dissemination might result from a very subtle (yet unknown) form of immunodeficiency. For example, it has been noted that a deficiency or mutation of the interferon-γ receptor 1 gene seems to increase susceptibility to mycobacterial infections [26]. In addition, the BCG strain used in Japan (Tokyo172 strain) is known to be very quickly killed in lysosomes after phagocytosis, which is the main reason for its high safety, and therefore, the clinical dose used in BCG vaccination usually causes almost no pathological changes (manufacturer's personal communication). Considering this information, the present infant might have had a defect in the phagocytosis and/or killing of the BCG. 

Table 2 lists 5 representative cases in which the outcome was fatal, taken from the recent literature for comparison (the present case is Case 6 in the table). Most of the cases involved miliary granulomas mainly in the lungs and liver. However, these lesions may even represent a natural immunoresponse to the vaccine [25], which is further supported by findings that similar dissemination occurred after intravesical BCG installation [3-8]. The alternative possibility is that this infant died due to an unrelated condition (*e.g.*, SIDS or asphyxia), with the granulomatous process representing a subtle pattern of BCG dissemination that might occur more commonly than is usually realized. In this sense, the present case may be a good example supporting this hypothesis.

**Table 2:** Six infants having granulomas found at autopsy

Case	Age (month)	Clinical situation	Autopsy findings
			Miliary granuloma
1	3	Sudden death	+ (lungs)
2*	2	Apneic bronchiolitis, GE reflux	+ (lungs), Acute superficial colitis
3*	2	Diarrhea, Seizures, Severe dehydration	+ (lungs and liver)
4*	5	Sudden death	+ (lungs, liver, and thyroid)
5	5	Chicken pox (found dead)	+ (lungs, liver, and kidneys)
6	5	GE reflux (found dead)	- (only liver granuloma)

Adapted from Ref.9. *PCR was positive for mycobacterium. The present case is Case 6.

There was no clinical and/or histopathological evidence suggesting an immunodisorder in these 6 infants.

The present case also raises two important questions regarding clinical pathology. One is whether the presence of epithelioid cell granulomas in the liver always indicates miliary tuberculosis. The answer is certainly "No"; the present infant had asymptomatic hepatic granuloma that was probably caused by BCG vaccination. The other one is why the granulomas formed only in the liver. Although we cannot answer this question, two possibilities can be considered: (1) BCG bacilli disseminated from the inoculation site, and reached organs showing affinity for this pathogen (such as the liver and spleen). Finally, the bacilli were phagocytosed by residual phagocytes. (2) BCG bacilli were phagocytosed at the inoculation site, and the phagocytes (macrophages) carrying the bacilli circulated throughout the whole body and reached a hub site for immunosurveillance (such as the liver), where they finally formed granulomas. In any event, it is well known that the liver is involved in many infectious diseases.

## Conclusion

The cause of the asymptomatic granulomas in this case was not identified, but BCG vaccination was considered the most likely.

## Abbreviations

BCG: bacillus Calmette-Guérin; GE: gastro-esophageal; H&E: hematoxylin and eosin; PCR: polymerase chain reaction; SIDS: sudden infant death syndrome.

## Consent

Written informed consent was obtained from the parents of the infant for publication of this case report and accompanying images. A copy of the written consent (written in Japanese with English translation) is available for review by the Editor-in-Chief of this journal.

## Competing interests

The authors declare that they have no competing interests.

## Authors' contributions

YT is the chief author. RT and TN assisted in the preparation of the manuscript. YK proofread the manuscript.

## References

[B1] Abramowsky C, Gonzalez B, Sorensen RU (1993). Disseminated bacillus Calmette-Guérin infections in patients with primary immunodeficiencies. Am J Clin Pathol.

[B2] Casanova J-L, Jouanguy E, Lamhamedi S, Blanche S, Fischer A (1995). Immunological conditions of children with BCG disseminated infection. Lancet.

[B3] Smith RL, Alexander RF, Aranda CP (1993). Pulmonary granulomata. A complication of intravesical administration of bacillus Calmette-Guérin for superficial bladder carcinoma. Cancer.

[B4] Palayew M, Briedis D, Libman M, Michel RP, Levy RD (1993). Disseminated infection after intravesical BCG immunotherapy. Detection of organisms in pulmonary tissue. Chest.

[B5] Proctor DD, Chopra S, Rubenstein SC, Jokela JA, Uhl L (1993). Mycobacteriemia and granulomatous hepatitis following initial intravesical bacillus Calmette-Guérin instillation for bladder carcinoma. Am J Gastroenterol.

[B6] McParland C, Cotton DJ, Gowda KS, Hoeppner VH, Martin WT, Weckworth PF (1992). Miliary *Mycobacterium bovis *induced intravesical bacillus Calmette-Guérin immunotherapy. Am Rev Respir Dis.

[B7] Le Mense GP, Strange C (1994). Granulomatous pneumonitis following intravesical BCG. What therapy is needed?. Chest.

[B8] Arzt MR, Forouhar F (1995). Granulomatous hepatitis as a complication of intravesical Bacillus Calmette-Guérin therapy for bladder carcinoma. Ann Clin Lab Sci.

[B9] Drut R, Quijano G (1998). Disseminated bacillus Calmette-Guérin, miliary type: autopsy findings and diagnosis using polymerase chain reaction. Pediatr Dev Pathol.

[B10] D'Amato RF, Wallman AA, Hochstein LH, Colaninno PM, Scardamaglia M, Ardila E, Ghouri M, Kim K, Patel RC, Miller A (1995). Rapid diagnosis of pulmonary tuberculosis by using Roche AMPLICOR *Mycobacterium tuberculosis *PCR test. J Clin Microbiol.

[B11] Pattishall EN, Kendig EL (1996). Sarcoidosis in children. Pediatr Pulmonol.

[B12] Colditz GA, Berkey CS, Mosteller F, Brewer TF, Wilson ME, Burdick E, Fineberg HV (1995). The efficacy of bacillus Calmette-Guérin vaccination of newborns and infants in the prevention of tuberculosis: meta-analysis of the published literature. Pediatrics.

[B13] Romanus V, Fasth A, Tordal P, Wiholm BE (1993). Adverse reactions in healthy and immunocompromised children under six years of age vaccinated with the Danish BCG vaccine, strain Copenhagen 1331: implications for the vaccination policy in Sweden. Acta Paediatr (Norway).

[B14] Casanova JL, Blanche S, Emile JF, Jouanguy E, Lamhamedi S, Altare F, Stéphan JL, Bernaudin F, Bordigoni P, Turck D, Lachaux A, Albertini M, Bourrillon A, Dommergues JP, Pocidalo MA, Le Deist F, Gaillard JL, Griscelli C, Fischer A (1996). Idiopathic disseminated bacillus Calmette-Guérin infection: a French national retrospective study. Pediatrics.

[B15] Esterly JR, Sturner WQ, Esterly NB, Widhorst DB (1971). Disseminated BCG in twin boys with presumed chronic granulomatous disease of childhood. Pediatrics.

[B16] Verronen P (1974). Presumed disseminated BCG in a boy with chronic granulomatous disease of childhood. Acta Paediatr Scand.

[B17] Catanzaro A, Melish ME, Minkoff DJ (1981). Disseminated BCG infection. J Pediatr.

[B18] Hogan LH, Macvilay K, Barger B, Co D, Malkovska I, Fennelly G, Sandor M (2001). *Mycobacterium bovis *strain bacillus Calmette-Guérin-induced liver granulomas contain a diverse TCR repertoire, but a monoclonal T cell population is sufficient for protective granuloma formation. J Immunol.

[B19] Rositto A, Molinaro L, Larralde M, Ranalletta M, Drut R (1996). Disseminated cutaneous eruption after BCG vaccination. Pediatr Dermatol.

[B20] Pedersen FK, Engbaek HC, Hertz H, Vergmann B (1978). Fatal BCG infection in an immunocompetent girl. Acta Paediatr Scand.

[B21] Trevenen CL, Pagtakhan RD (1982). Disseminated tuberculoid lesions in infants following BCG vaccination. Can Med Assoc J.

[B22] Katzir Z, Okon E, Ludmirski A, Sherman Y, Haas H (1984). Generalized lymphadenitis following BCG vaccination in an immunocompetent 12-year-old boy. Eur J Pediatr.

[B23] Lechaux A, Descos B, Mertani A, Souillet G, Gilly J, Hermier M (1986). Infection généralisée a BCG d'évolution favorable chez un nourrison de 3 mois sans déficit immunitaire reconnu. Arch Fr Pediatr.

[B24] Kroger L, Brander E, Korppl M, Wasz-Hockert O, Backman A, Kroger H, Launiala K, Katila ML (1994). Osteitis after newborn vaccination with three different Bacillus Calmette-Guérin vaccines: twenty-nine years of experience. Pediatr Infect Dis J.

[B25] Gormsen H (1956). On the occurrence of epithelioid cell granulomas in the organs of BCG-vaccinated human beings. Acta Pathol Microbiol Scand, Suppl 111.

[B26] Jouanguy E, Altare F, Lamhamedi S, Revy P, Emile JF, Newport M, Levin M, Blanche S, Sebun E, Fischer A, Casanova JL (1996). Interferon-γ-receptor deficiency in an infant with fatal bacille Calmette-Guérin infection. New Engl J Med.

